# Lentiviral in situ targeting of stem cells in unperturbed intestinal epithelium

**DOI:** 10.1186/s12915-022-01466-1

**Published:** 2023-01-11

**Authors:** George B. Garside, Madeline Sandoval, Slobodan Beronja, K. Lenhard Rudolph

**Affiliations:** 1 Leibniz Institute on Aging, Fritz Lipmann Institute, 07745 Jena, Germany; 2 National Center for Tumor Diseases, 01307 Dresden, Germany; 3 Division of Human Biology, Fred Hutchinson Cancer Center, Seattle, WA 98109 USA

**Keywords:** Genetic engineering, Microinjection, Intestine, APC, Transduction, Intestinal stem cells, Endoderm, Clonality, Crypt formation, Stable in vivo targeting

## Abstract

**Background:**

Methods for the long-term in situ transduction of the unperturbed murine intestinal epithelium have not been developed in past research. Such a method could speed up functional studies and screens to identify genetic factors influencing intestinal epithelium biology. Here, we developed an efficient method achieving this long-sought goal.

**Results:**

We used ultrasound-guided microinjections to transduce the embryonic endoderm at day 8 (E8.0) in utero. The injection procedure can be completed in 20 min and had a 100% survival rate. By injecting a small volume (0.1–0.2 μl) of concentrated virus, single shRNA constructs as well as lentiviral libraries can successfully be transduced. The new method stably and reproducibly targets adult intestinal epithelium, as well as other endoderm-derived organs such as the lungs, pancreas, liver, stomach, and bladder. Postnatal analysis of young adult mice indicates that single transduced cells at E8.0 gave rise to crypt fields that were comprised of 20–30 neighbouring crypts per crypt-field at 90 days after birth. Lentiviral targeting of *Apc*^Min/+^ mutant and wildtype mice revealed that heterozygous loss of *Apc* function suppresses the developmental normal growth pattern of intestinal crypt fields. This suppression of crypt field sizes did not involve a reduction of the crypt number per field, indicating that heterozygous *Apc* loss impaired the growth of individual crypts within the fields. Lentiviral-mediated shRNA knockdown of p53 led to an approximately 20% increase of individual crypts per field in both *Apc*^+/+^ and *Apc*^Min/+^ mice, associating with an increase in crypt size in *Apc*^Min/+^ mice but a slight reduction in crypt size in *Apc*^+/+^ mice. Overall, p53 knockdown rescued the reduction in crypt field size in *Apc*-mutant mice but had no effect on crypt field size in wildtype mice.

**Conclusions:**

This study develops a novel technique enabling robust and reproducible in vivo targeting of intestinal stem cells in situ in the unperturbed intestinal epithelium across different regions of the intestine. In vivo somatic gene editing and genetic screening of lentiviral libraries has the potential to speed up discoveries and mechanistic understanding of genetic pathways controlling the biology of the intestinal epithelium during development and postnatal life. The here developed method enables such approaches.

**Supplementary Information:**

The online version contains supplementary material available at 10.1186/s12915-022-01466-1.

## Background

The intestinal epithelium is the most rapidly self-renewing tissue in adult mammals and serves to absorb nutrients whilst providing protection against environmental insults. For these reasons, the intestine is an intensively studied organ across multiple model organisms, including mice. There have been multiple efforts to establish a reliable method that would enable researchers to genetically target the mammalian intestinal epithelium. Stable targeting of the intestinal epithelium requires transduction and stable integration of the transgene into the genome of intestinal stem cells (ISCs). Due to the anatomic location of ISCs in basal crypts (hidden deep in the intestinal epithelium of adult mice), it was until now impossible to stably target the unperturbed intestinal epithelium throughout all intestinal regions in vivo.

Current methods of intestinal epithelium transduction use lentiviruses or retroviruses to first permanently transduce intestinal organoids in culture, which are then transplanted onto the native intestinal epithelium or injected into the caecal wall/renal capsule of immunodeficient mice [1–4]. These methods of transduction have several shortcomings. Specifically, both methods require that organoids must first be expanded in vitro and transduced with the vector of choice. In addition, ectopically transplanted organoids lack the native intestinal microenvironment and must be first transformed to grow independently [2]. In addition, rectal engraftment of targeted organoids requires epithelial abrasion [1]. The method is also physically limited to the lower colon and possesses a low transduction efficiency (engraftment area 1 ± 0.7 in total mm^2^) [1].

Direct in situ targeting of the unperturbed intestinal epithelium would have the advantage to exclude selection processes that may occur during ex vivo culture expansion and the transplant engraftment of genetically altered cells. Here, we developed an efficient method for long-term in situ targeting of ISCs in the unperturbed, non-immunocompromised, intestinal epithelium of wildtype mice.

## Results

In the first strategy to target the mouse intestine, we explored in utero injections of lentivirus that targeted the amniotic fluid surrounding mouse embryos. This method was successful in previous studies in targeting the skin epidermal stem cells when injections were conducted at E9.5 of development [5]. Here, we used the same methodology at different timepoints of embryonic development, ranging from E14.0 to E18.0. The rational for this approach was that embryonic swallowing of the amniotic fluid could lead to targeting of the mouse intestine [6, 7]. However, whilst lentiviral injection of the amniotic fluid was successful and swallowing of amniotic fluid was clearly detectable after injection of fluorescent beads, we did not observe any intestinal targeting at birth after lentiviral injection (*n* = 51). It is possible that villus formation, which is known to occur at E14.5 [8], may have prohibited efficient targeting of ISCs. An alternative method of directly targeting the early intestinal lumen between E9.0 and E12.0 with lentivirus also did not result in successful transduction (*n* = 78), likely due to the small target space.

**Fig. 1:**
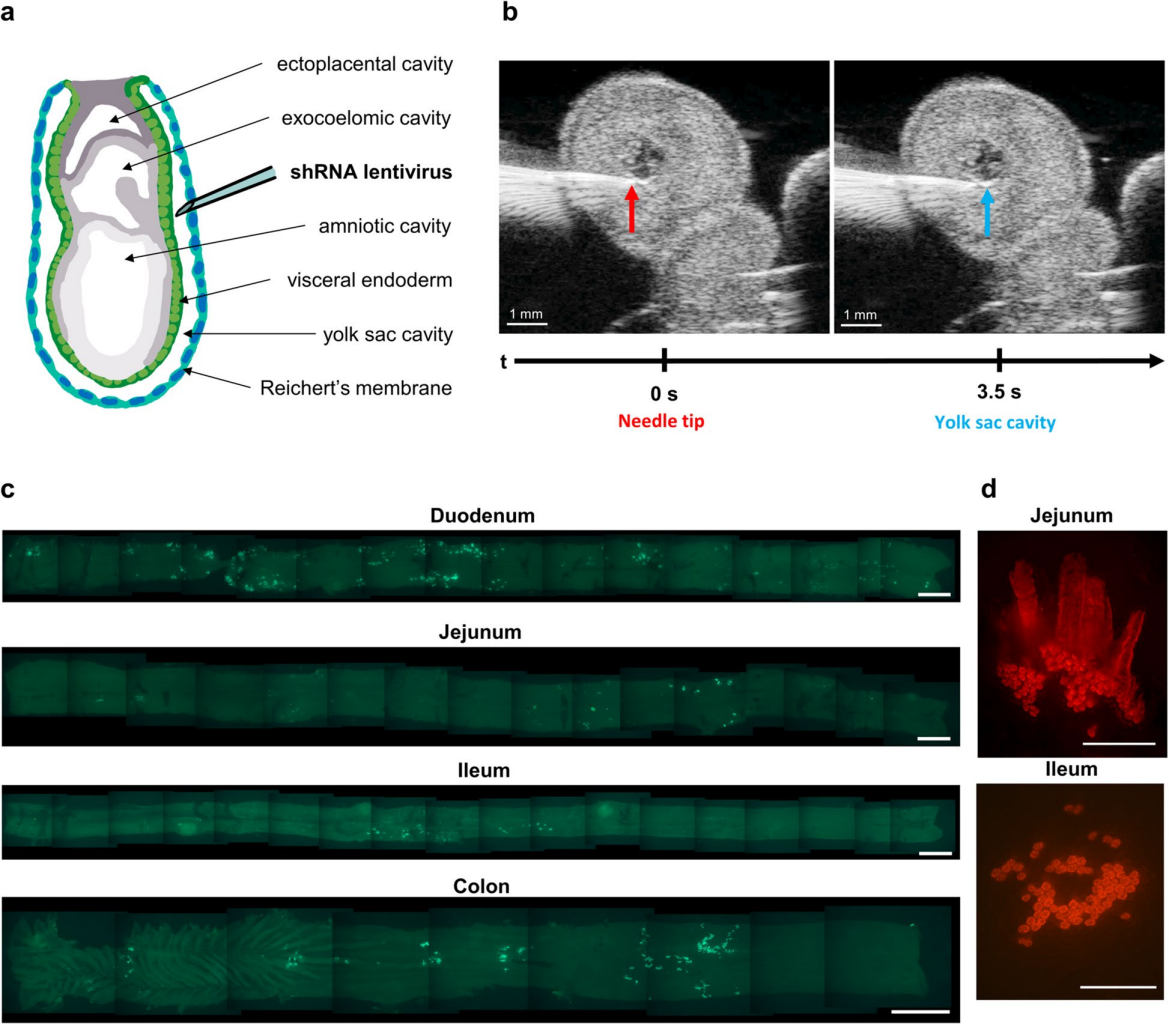
Lentiviral injection of E8.0 mouse embryos achieves stable targeting of intestinal epithelium in adult mice. **a** Schematic diagram of the egg cylinder at embryonic days 7.75–8.0. Figure adapted from Ermakova et al. [9]. **b** Targeting of the endoderm on embryonic day 8 (E8.0) was achieved by ultrasound-guided microinjections as indicated by 4-s time-lapse images (see Suppl. Movie). A needle comes from the left piercing the embryo (centre frame) attached to a uterine horn. Silicone support (right) was used to stabilize the uterine horn for injections. Left: At 0 s, the embryo has been pierced and the needle tip (red arrow) rests in the small yolk sac cavity in an E8.0 embryo. Right: At 3.5 s, 69 nl of concentrated lentivirus has been injected into the yolk sac cavity leading to a small expansion of the yolk sac cavity (blue arrow). Images were extracted from the supplemental movie and cropped to highlight injection and embryo. **c** Example of the composite image analysis of transduced crypt fields from a luciferase (GFP)-infected mouse. Mouse embryos were injected at E8.0, injected mice were analysed at postnatal day 90. Scale bars: 5 mm. **d** Example fluorescent stereoscope images of small intestine crypt fields after transducing mice at E8.0 with an H2B-RFP lentiviral construct, resulting in nuclear red fluorescence staining of the transduced crypts. Crypt fields were defined as > 10 transduced crypts in direct contact or being in close proximity (< 5 crypts distance). Top: low magnification Z-stack image highlighting transduced jejunal crypts with associated RFP-positive villi. Bottom: high-magnification image of H2B-RFP transduced ileal field, with multiple individual transduced crypts visible. Scale bars: 500 μm

To overcome these hurdles, a completely new strategy was developed whereby lentiviral injections were carried out at E8.0 prior to primitive intestinal formation. At this stage of embryonic development, the endoderm is exposed on the outside of the embryo and encapsulated by Reichert’s membrane (Fig. 1a); by E9.0, it invaginates and goes on to form a closed loop (the primitive intestine) [9, 10]. To directly target the endoderm at the E8.0 stage (before invagination of the intestine), the needle was ultrasound-guided in between the endoderm and the Reichert’s membrane, followed by injection of a small volume (0.1–0.2 μl) of concentrated lentivirus into the yolk-sac cavity at that position (Fig. 1b and Suppl. Movie). Strikingly, the new technique intermittently transduced the entire small and large intestine in adult C57BL6/6J mice, an area of 9.9 ± 4.3mm^2^ on average per mouse. In adult mice, targeting of intestinal epithelium at E8.0 resulted in fields of transduced crypts and associated villi, which were surrounded by untransduced tissue (Fig. 1c, d). The transduced adult intestine at 3 months presented with dispersed fields of transduced crypts throughout the small intestine and colon (Fig. 1c, Suppl. Fig. 1).

C57BL/6J mice had an average of 0.75 ± 0.9% (max. 5.6%, Suppl. Fig. 2) of their intestinal epithelium targeted using an average lentiviral titre of 8 × 10^7^ TU/ml, with the inter-group (different mothers) standard deviation for the transduction area of the intestine only slightly higher than the intra-group (siblings) variation (0.9% vs 0.7% respectively). This transduction produced a speckled appearance of fluorescent crypt fields throughout the intestine with large regions of untransduced tissue in between. Mice were counted as ‘successfully transduced’ if two distinct regions of their intestines exhibited clear fluorescence (either duodenum, jejunum, ileum, or colon).

In line with previous data on crypt fixation [11], we observed a mottled appearance of crypts at E17.0-E18.0, which turned into visibly solid, contiguous transduced fields by postnatal day (P14) (Suppl. Fig. 3). At P14 and later, the transduced fields often appeared fragmented, including nearby ‘satellite’ transduced crypts (Fig. 1d, Suppl. Fig. 3). It is possible that the formation of ‘satellite’ crypts was due to normal crypt fission (splitting, known to take place during postnatal crypt fixation and growth) occurring in both transduced and untransduced crypts. The ‘satellite’ appearance of transduced fields may have originated by migration of untransduced crypts into the transduced crypt field. According to this model, the observed crypt fields (including satellites) could represent the outgrowth of single cells initially transduced on E8.0. However, it cannot be excluded that satellite crypts and contiguous crypt fields may also derive from different targeting events co-occurring in regionally confined pockets of the transduced gut anlage at E8.0. To answer this question, additional experiments would have to be conducted, such as lentiviral infections with barcoded libraries in order to determine the clonality of individual crypt fields (representing one or more clones).

**Fig. 2:**
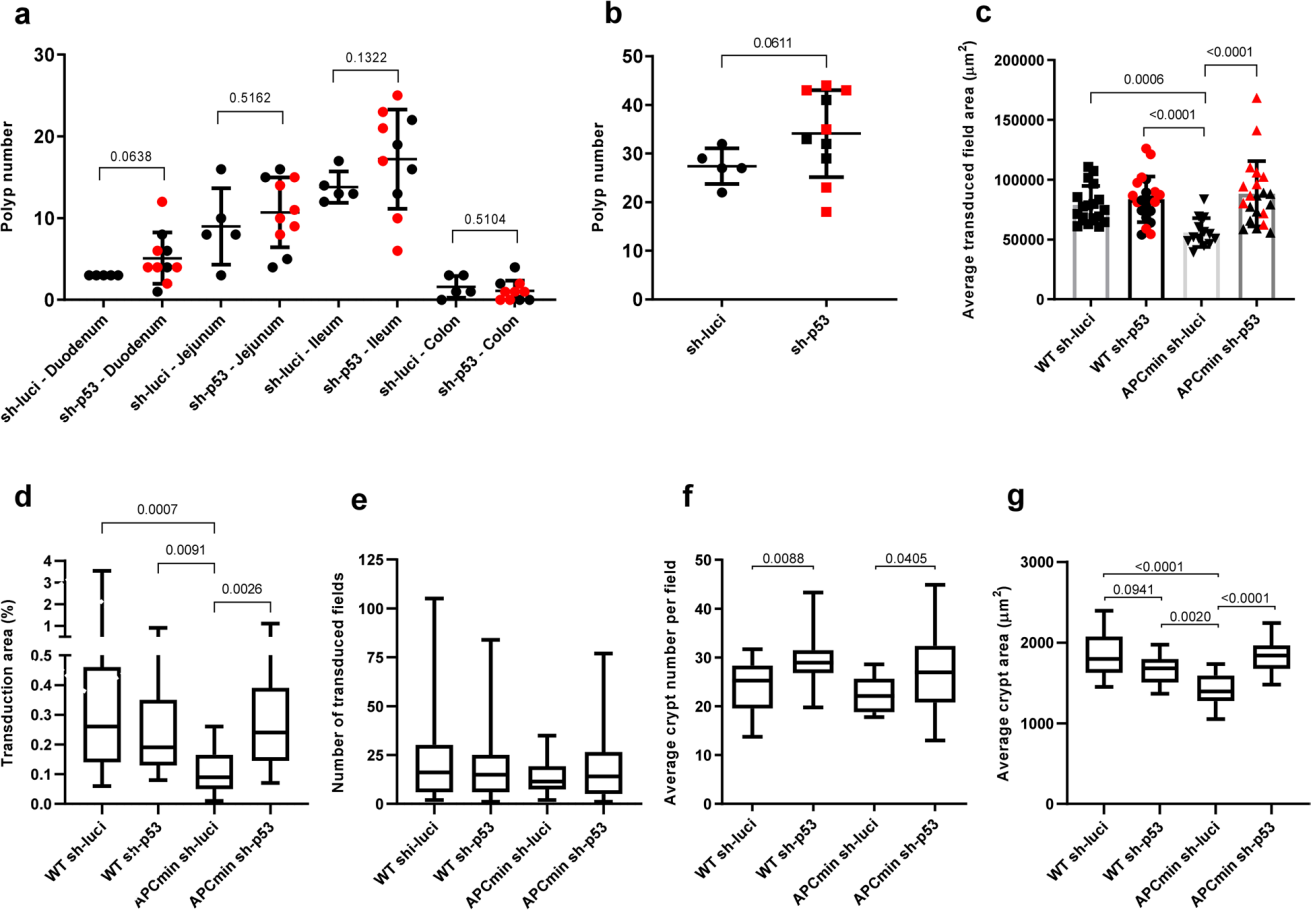
90-days-old *Apc*^Min/+^ and *Apc*^+/+^ mice were lentivirally targeted on E8.0 with p53 or luciferase shRNA. **a**, **b** Intestinal polyp numbers in mice targeted with the indicated shRNAs. Two shRNAs were used for p53 knockdown (sh1558 in black and sh852 in red). **a** The indicated regions of the intestine were analysed side by side with Welch’s *t*-test without multiple comparison testing. **b** The total polyp number in the whole intestine was determined and analysed by Welch’s *t*-test. **a, b** sh-luci and sh-p53 polyp numbers in the different intestinal regions were pooled for normality testing and were found to be normally distributed (D’Agostino and Pearson normality test). *Apc*^Min/+^ mice: luciferase *n* = 5, p53 sh842 *n* = 6, p53 sh1558 *n* = 4; *Apc*^+/+^ mice: luciferase *n* = 8, H2B-RFP *n* = 3, p53 sh842 *n* = 5, p53 sh1558 *n* = 5. **c** Mean transduced crypt field area of entire small intestine (duodenum, jejunum and ileum regions combined) transduced with sh-p53 or control sh-luciferase. *Apc*^+/+^ regions: sh-luciferase *n* = 18, sh-p53 *n* = 21; *Apc*^Min/+^ regions: sh-luciferase *n* = 13, sh-p53 *n* = 21. **d** The transduced area of the entire small intestine from WT or *Apc*^Min/+^ mice transduced with sh-p53 or control sh-luciferase. *Apc*^+/+^ regions: sh-luciferase *n* = 19, sh-p53 *n* = 21; *Apc*^Min/+^ regions: sh-luciferase *n* = 13, sh-p53 *n* = 21. **e** Number of transduced crypt fields present in the entire small intestine from WT or *Apc*^Min/+^ mice transduced with p53 or control luciferase shRNA. No significant differences were found. *Apc*^+/+^ regions: sh-luciferase *n* = 26, sh-p53 *n* = 27; *Apc*^Min/+^ regions: sh-luciferase *n* = 14; *Apc*^Min/+^ sh-p53 *n* = 29. **f** Mean average crypt number per field present in the entire small intestine. *Apc*^+/+^ regions: sh-luciferase *n* = 22, sh-p53 *n* = 22; *Apc*^Min/+^ regions: sh-luciferase *n* = 12, sh-p53 *n* = 22. **g** Mean average crypt area present in the whole small intestine. *Apc*^+/+^ regions: sh-luciferase *n* = 15, sh-p53 *n* = 18; *Apc*^Min/+^ mice: sh-luciferase *n* = 14, sh-p53 *n* = 18. **c**–**g** Data were log_2_ transformed and analysed with two-way ANOVA and Tukey’s multiple comparisons test—untransformed data plotted

To explore the potential of the newly developed method for the analysis of gene interaction studies in transgenic mice, we explored whether the knockdown of *Trp53* compared to a luciferase targeting control shRNA would impact on the growth of polyps or transduced crypt foci in *Apc*^+/+^ mice *versus Apc*^Min/+^ mice (for knockdown efficiency of the *Trp53*-shRNAs see Suppl. Fig. 4). *Apc*^Min/+^ mice carry a heterozygous *Apc* mutation [12], which abrogates gene function and leads to adenomatous polyposis in both mice and humans by loss of heterozygosity [13]. In regard to macroscopic polyp numbers, there was only a non-significant trend towards elevated levels of polyps in sh-p53 transduced mice compared to control shRNA transduced mice in some regions of the intestine (Fig. 2a) as well as in the total numbers of polyps across the entire intestine (Fig. 2b, *p* = 0.0611).

The number of fully transduced polyps was overall too low to assess a direct role of p53 on polyp formation (Suppl. Fig. 5). The comparisons made on the effects of lentivirus transduction on polyp number/labelling in *Apc*^Min/+^ mice have to be viewed as preliminary data as both transduction and polyp formation are rare stochastic processes. In the literature, discrepant results have been reported on possible effects of p53 gene status on polyp formation in *Apc*^Min/+^ mutant mice [14–18].

Interestingly, *Apc*^Min^ gene status had a significant impact on crypt field development, which was abrogated by p53 inhibition. Specifically, the average size of transduced intestinal crypt fields was significantly smaller in *Apc*^Min/+^ mice compared to *Apc*^+/+^ mice (Fig. 2c, *p* = 0.0006). Whilst sh-p53 infection had no impact on crypt field area size in *Apc*^+/+^ mice, p53 knockdown abrogated the suppression in crypt field size in *Apc*^Min/+^ mice (Fig. 2c, *p* < 0.0001). These results correlated with similar changes in the total area of transduced small intestine (Fig. 2d), despite an equal level of transduced crypt fields (Fig. 2e). When the crypt number per field was analysed (Fig. 2f), it revealed an equivalent number of control (sh-luci) targeted crypts in *Apc*^Min/+^ and *Apc*^+/+^ mice, suggesting that the *Apc*^Min^ mutation may constrain crypt field area size by reducing the size of individual crypts. Indeed, an analysis of individual crypt area size (Fig. 2g) revealed significantly reduced individual crypt sizes between sh-luci targeted *Apc*^Min/+^ and *Apc*^+/+^ mice (*p* < 0.0001). p53-knockdown was found to increase the crypt number independent of the *Apc* genotype (Fig. 2f). Of note, p53-knockdown increased average individual crypt size in *Apc*^Min/+^ mice but not in *Apc*^+/+^ mice (Fig. 2g), thus resulting in a rescue of the reduction in crypt size in *Apc*^Min/+^ mice (Fig. 2c).

Together, the effect of *Apc*^Min^ on crypt growth can be assumed to be at least partially p53-dependent, since p53-knockdown abrogated the suppressive effect of the heterozygous *Apc*^Min^ mutation on crypt field size (Fig. 2c, average field area of 55771 μm^2^ in *Apc*^Min/+^ sh-luci vs 88268 μm^2^ *Apc*^Min/+^ sh-p53) and individual crypt size (Fig. 2g, average crypt size of 1405 μm^2^ in *Apc*^Min/+^ sh-luci vs 1835 μm^2^ in *Apc*^Min/+^ sh-p53). In addition, p53-knockdown had an *Apc* genotype-independent effect on increasing the crypt numbers per field (Fig. 2f).

## Discussion

Together, this study has developed a robust method enabling stable targeting of intestinal stem cells and the intestinal epithelium by lentiviral transduction of the unperturbed intestinal epithelium during early development. The study provides a preliminary set of data on lentiviral intestinal targeting of *Apc*^Min/+^ mutant versus wildtype mice. These experiments provide experimental evidence (i) that this method can label crypt fields that are known to originate from single cells during development, (ii) that the size of crypt fields is reduced in *Apc*^Min/+^ mutant mice compared to wildtype mice, and (iii) that the method can achieve an in vivo knockdown of target genes by lentiviral shRNA transfer, which enabled us to show that *Apc*^*Min*^ mutation mediated suppression of crypt field growth is p53-dependent. Whilst the mechanistic nature of these observations remains largely unclear, the observations nicely illustrate the potential of the newly developed method.

As the shRNAs used in this study are transferred by lentiviral vectors and were constitutively expressed, we cannot conclude at what specific stage of embryonic development or postnatal development the suppression of crypt field growth occurs in *Apc*^Min/+^ mice. To address this point would require the usage of inducible systems, such as tetracycline [19, 20] or tamoxifen [21] inducible mouse models. It remains to be investigated what mechanisms lead to inhibition of the growth of individual crypts within a crypt field in *Apc*^Min/+^ mice compared to *Apc*^+/+^ mice. This study implies that the effect is at least partially p53-dependent. It is possible that Wnt-activation in *Apc*^Min^ mutant mice enhances induction of p53-dependent DNA damage signals in intestinal crypts. Previous studies have delineated a role of Wnt-pathway activation in enhancing p53-dependent DNA damage response in intestinal stem cells [22]. It is conceivable that Wnt-activation in heterozygous *Apc*^*Min/+*^ mice triggers p53 activation, leading to a reduced growth of crypts within a crypt field thus limiting overall crypt field size, which in turn may be restored to normal size by *p53*-depletion. Alternatively, *Apc*^Min/+^ may enhance mechanisms of confluency-induced growth arrest of cells, which are known to be also *p53*-dependent [23, 24].

Together, this study demonstrates a new microinjection technique that enables robust, reproducible, and stable in vivo targeting of intestinal stem cells in situ in the unperturbed intestinal epithelium. The technique enables researchers to genetically target ISCs and the intestinal epithelium in a physiological in vivo setting. The new technique is capable of transducing all regions of the gastrointestinal tract including stomach, small intestine, and colon. Of note, this technique also targets other endoderm-derived organs (Suppl. Fig. 6) such as the pancreas, liver, stomach, bladder, and lung, and therefore may be of use to other fields of research. Organ specificity should also be achievable by infecting genetically modified mice that activate the lentiviral constructs in a cell type and/or organ specific manner.

## Conclusions

This study demonstrates a new method for stable targeting of unperturbed intestinal epithelium during early development. We provide a show case on *Apc*^Min^-induced growth restriction of intestinal crypts during development leading to a reduced size of intestinal crypt fields in young adult mice. This phenotype can be rescued by lentiviral targeting of ISC at day E8.0 of development with p53-shRNAs implying that *Apc*^Min^-induced growth restriction of intestinal crypts and crypt fields is p53 dependent. These results demonstrate that the technique can be employed to study cell and cell field kinetics during the development of the intestine and genetic interaction involved in the regulation of such processes. The new technique could also be used to investigate the influence of genes on cancer formation, ageing, and microbiota-host interactions. The great advantage of this method is that it enables the conductance of genetic in vivo screens in the unperturbed intestinal epithelium of mice and possibly other species. As such, the current method development represents a major breakthrough for the field and will speed up discoveries on intestinal epithelium biology.

## Methods

### Mice

Eight-week-old female C57BL/6J (C57BL/6J; Stock #000664) and *Apc*^Min/+^ (C57BL/6J-Apc^Min^/J; Stock #002020) male mice were obtained from The Jackson Laboratory for subsequent breeding and in utero microinjection experiments. Male and female experimental *Apc*^Min/+^ mice were killed at or about 90 days old. Animals were always provided a minimum 1-week period of acclimatization before any experimentation or breeding was commenced. No a priori criteria were explicitly set for animal exclusion; all animals maintained good health and none were excluded from analysis. No randomisation mechanism was used to allocate mice into shRNA groups. Group allocation was performed by GBG, with ImageJ analysis performed in a blind manner.

All animal experiments were conducted in accordance with ethical regulations of the Fred Hutchinson Cancer Center and IACUC-approved protocols (project licence number 50814). At least once a day the condition of the animals was examined by direct inspection by the animal caretakers. Mice were to be euthanized if they ever met the humane endpoint. Adult mice were euthanized by carbon dioxide (CO_2_) asphyxiation, followed by confirmatory cervical dislocation. Pups < P21 and foetuses were killed via decapitation.

All animals were housed in centralized facilities at the Fred Hutchinson Cancer Center under the care and supervision of the Comparative Medicine Unit (CM). The animal facility is managed in compliance with the Guide for the Care and Use of Laboratory Animals, 2011. The FHCC is fully accredited by the Association for Assessment and Accreditation of Laboratory Animal Care, International (AAALAC) assured by NIH Office of Animal Welfare (OLAW) and registered as a research facility with the USDA. Veterinary services are provided by a staff of 5 on-site veterinarians.

All animals are housed in individually ventilated and HEPA-filtered microisolator cage environments (Allentown Inc. & Tecniplast) that are autoclaved prior to use. Mice were housed in the same room and rack to minimize location as a confounding factor. All animal feed and cage enrichment material were sterilized and only purified water was provided. Mice are given ad libitum access to food and water. Husbandry was done in three-sided, height-adjustable HEPA filtered laminar flow cabinets using appropriate disinfectants. Mice were kept on a 12-h day-night cycle. Mice were individually housed post-microinjection surgery and provided translucent, red plastic shelters and supplemental nesting material. Mice were assessed every day for 5 days following surgery and monitored closely until birth.

### Ultrasound pregnancy check

Pregnancy was confirmed using ultrasound (Vevo 2100). Mothers were mated for 1–2 weeks and checked for pregnancy at embryonic days 6–7 (E6.0-E7.0) and thereafter every few days as appropriate. The embryonic stage could be accurately determined from E6.0 onwards and was additionally confirmed on the day of the microinjection surgeries (typically E8.0—see results). Mothers were anesthetized using an isoflurane vaporizer set to 2.5–3.0% with the oxygen regulator set to 1 l/min; depilatory cream was then used to remove abdominal hair from a 2 × 2 cm area using a cotton-tipped applicator. The area was then cleaned with 70% ethanol and ultrasound gel applied for imaging. Once the mice were imaged, they were returned to their cages for recovery.

### Microinjection needle preparation

Needles (Drummond 3.5″ borosilicate capillaries) were pulled to a taper using a Sutter P-87 micropipette puller using the following variables: heat = 769; velocity = 140; time = 100; pull = 0; pressure = 200.

Once pulled, the needle tip was snapped off using fine tip forceps at the level where its diameter was ∼30 μm. The needle tip was bevelled at 25° on a fine-grade abrasive plate (Narishige model EG 44) with regular wetting for 10 min. Afterwards, the needle tip was microscopically checked under × 10 magnification to ensure a clean bevel without deformities.

Once bevelled, a 26G 1/2 needle and syringe were used to push distilled water through the needle in order to remove any debris that may have accumulated during the sharpening process. Finally, the needle was sterilized by pushing through 70% ethanol, before expelling all the liquid and being left to air dry. The dry needle can then be used immediately or stored indefinitely.

### Loading of needle for microinjection

A prepared needle was backfilled with mineral oil using a 26G 1/2 needle, ensuring there were no bubbles along its length. The oil-filled needle was then loaded onto the Nanoinject II system (Drummond).

Mineral oil was expelled from the needle by extending the piston to its maximum position; a small volume of oil remained in the needle to prevent the lentivirus payload from contacting the piston.

A small volume (10 μl) of concentrated lentivirus was dispensed onto a hydrophobic surface such as parafilm. The needle tip was then lowered into the middle of the drop, and the piston slowly retracted to its minimum position. This will load ~5 μl of virus. Once the needle was loaded with lentivirus, the needle tip was lowered into a dish filled with sterile PBS to prevent the virus at the tip from drying out and potentially clogging the needle.

### Ultrasound-guided embryonic microinjection

The microinjection injection technique used to target the endoderm was the same as that published by Beronja and Fuchs [5] to target the skin. The steps below were taken from Beronja and Fuchs 2013 and are reproduced below with minor modifications [25]. The technique was modified at the targeting step (#13) for embryonic day 8 (E8.0) endoderm targeting.

1. The Vevo 2100 ultrasound imaging system (VisualSonics) and heating platform (37 °C) are turned on.
2. The modified Petri dish was constructed (beforehand): (a) the backing from one side of the double sided membrane tape was removed and stuck to the bottom of the Petri dish so that it evenly surrounds the central opening; (b) the remaining side of the double-sided membrane tape was removed; (c) a square of silicone membrane was placed over the exposed membrane tape and pressed firmly down to ensure tight adhesion and to remove any air pockets; (d) using micro-dissecting scissors, a small rectangular opening was cut longitudinally in the silicone membrane measuring 2 mm (width) × 10 mm (length).
3. A pregnant mouse was anesthetized within the induction chamber by setting the oxygen regulator to 1 l/min and isoflurane vaporizer to 2.5–3.0%. After ∼3 min, the animal was checked to determine if it was anesthetized by performing the paw pinch test. It is important to note that an animal’s age and genetic background may influence the sensitivity to anaesthesia.
4. Once fully anesthetized, the oxygen/isoflurane flow from the induction chamber was switched to the nose cone attached to the heated animal platform. The isoflurane vaporizer rate may be lowered, if necessary, for the surgery (i.e. to 2%). The anesthetized mouse was then removed from the induction chamber and placed ventral side up (supine) onto the heated animal platform (37 °C). Eye cream was applied to prevent drying (e.g. Bepanthen).
5. The mouse was immobilized by taping its hind legs to the animal platform using surgical tape.
6. A 2 × 2 cm area of abdominal hair was removed using depilatory cream and a cotton-tipped applicator. The process may be aided by gentle, continuous circular rubbing with the applicator. Once the hair was sufficiently removed, and any remaining depilatory cream wiped away, the area was cleaned with 70% ethanol. The abdomen was then scanned to stage the pregnancy—timepoints before E6 may prove difficult to accurately assess.
7. The abdomen was then opened by pinching and lifting up the abdominal skin to make a ∼2 cm incision along the midline of the mouse, being careful not to damage any of the underlying structures. Sharp micro-dissecting scissors were used to cut a similarly sized incision along the poorly vascularized peritoneal midline (linea alba).
8. The total number of embryos may be counted at this point on the left and right uterine horns. Blunt tip forceps were used to gently remove one of the horns, griping lightly between the embryo implantation sites to expose a segment of 3–4 embryos.
9. Four cubes of modelling clay were positioned around the mouse to support the modified Petri dish. Simultaneously, the dish was lowered gently over the exposed uterine segment whilst an exposed segment of ~3–4 embryos was carefully pulled through into the dish using blunt tipped forceps. The dish was then levelled and stabilized by pressing it into the modelling clay.
10. The semi-circular silicone plug was placed on the right side of the dish to steady and support the exposed embryos against the force of injections. The modified Petri dish was filled with sterile, room temperature PBS. The silicone membrane of the modified Petri dish prevents leaking by adhering to the mother’s abdominal skin.
11. Ensuring the ultrasound scan head is correctly housed in the holder with a 30° upward angle to facilitate injections, the scan head was lowered into the PBS and the animal platform adjusted to visualize a single embryo.
12. The injection apparatus was brought towards the animal platform and the needle positioned ~1 cm short from the embryo in the modified Petri dish. The needle was moved closer to the embryo using the micromanipulator so that the tip appears on the ultrasound (within ~5 mm). The needle was then brought into plane—where the tip appears the brightest.
13. Next all embryos of the uterine horn were sequentially injected. The injection dial was used to move the needle into contact with the uterine wall. For E8 endoderm targeting the needle was gently but firmly pushed forward until it punctured and passed through Reichert’s membrane, with the needle tip positioned in contact with the exposed visceral endoderm (Fig. 1b). The Nanoinjector ‘inject’ button was used to dispense x2 69 nl volumes of lentivirus into the yolk sac cavity (Suppl. Movie).
14. The remaining embryos from the entire section of the uterine horn were injected. Once complete, the scan head and silicone plug from the modified Petri dish were removed and the PBS aspirated. The exposed embryos were carefully returned to the abdominal cavity using a cotton-tipped applicator and the dish removed. The remaining embryos were then sequentially injected using the same procedure, with the aim to keep the surgery under 30 min.
15. Once the desired number of embryos was injected, the abdominal area was cleaned with lint-free tissue paper to remove any PBS that may have leaked into the abdominal cavity. The peritoneal incision was then closed using absorbable sutures, and the abdominal skin closed with staples (two staples were usually sufficient).
16. An insulin syringe was used to administer a subcutaneous injection of 0.03 cc of Buprenorphine (Buprenex) or other analgesic at recommended dose.
17. The mouse was returned to a heated recovery cage and monitored until fully recovered (~10 min).

During the embryo transductions conducted according to the above describe new methodology, all mothers survived and tolerated the procedure well (*n* = 25), with an average litter size of 5.6 at weaning in mothers with surviving litters (*n* = 23) and 4.8 when including two litters in which no pups survived (*n* = 25). The average litter size in mothers with surviving litters is comparable to the 5.5 reported for the C57BL/6J substrain [26]. Within a litter, an average of 31 ± 26% of pups were transduced (*n* = 111, Suppl. Table 1).

### High titre lentiviral production for in utero injections

Typically for each viral construct 2 × 500 cm^2^ plates of 293FT packaging cells were prepared (yielding ~140 ml of viral supernatant, which was concentrated ~2000x to 70 μl of high titre virus for in utero injections). 500 cm^2^ plates were coated with poly-L-lysine stock (Sigma P4832-50ML), diluted 1:10 in PBS with 50 ml used for each plate for 1 h at room temperature. After 1 h, the poly-L-lysine solution was removed, and the plate rinsed 3 times with PBS.

For two 500 cm^2^ plates 275 μg of pLKO.1 H2B-RFP (or vector of choice), plus 275 μg of the packaging plasmid pPAX2 and 180 μg of plasmid pMD2.G were used. All plasmids were prepared using Qiagen’s Endotoxin Free Maxiprep kit. 293FT cells were cultured at low passage (<P20) and not allowed to become confluent whilst being subcultured. 500 μg/ml G418 (Geneticin) was used in the culture medium until transfection in order to maintain the expression of the SV40 large T-antigen.

Plates were transfected when cells were ~65–75% confluent using the calcium phosphate method. For 2 × 500 cm^2^ plates, 165 ml pre-warmed D10 medium (D10 DMEM, FBS (10% v/v), Pen/Strep/L-glut mix (1% v/v), 100 mM Sodium Pyruvate (1% v/v), 7.5% Sodium Bicarbonate (1% v/v), G418) was added to a disposable PETG 250 ml media bottle (Nalgene # 342020-0250). In a 50 ml conical tube, 275 μg pVector, 275 μg pPAX2 and 180 μg pMD2.G were mixed together. 2.28 ml room temperature 2M CaCl_2_ was added, with sterile distilled water added to make a total volume of 9.5 ml, then inverted several times to mix. 9.5 ml 2x HBS was added and mixed by inverting 4 times and incubated at RT for exactly 60 s. The transfection mixture was added to the bottle of pre-warmed D10 and mixed. The media was aspirated from the 500 cm^2^ plates and 90 ml transfection/media mix was slowly added to the side of each plate. Plates were incubated in a 37 °C/7.5% CO_2_ incubator overnight.

Twelve to fourteen hours post-transfection, transfection medium was removed and the plates rinsed once with pre-warmed D10. Plates then had 70 ml of fresh viral production medium (VPM - UltraCulture, Pen/Strep/L-glut mix (1% v/v), 100 mM sodium pyruvate (1% v/v), 7.5% sodium bicarbonate (1% v/v), 0.5M sodium butyrate (1% v/v)) added to each plate.

Sixty-four hours post-transfection, the viral supernatant was collected (48 h after adding VPM) and filtered using 0.45 μM Millipore low-protein binding filter units (SCHVU02RE; Millipore).

### Concentration of lentivirus

First, the supernatant was filtered using low speed centrifugation through a 100 kDa MW cut-off Millipore Centricon 70 Plus cartridge (Merck; UFC710008) to concentrate ~140 ml of viral supernatant to < 1 ml. Centricon 70 Plus cartridges were pre-rinsed with 10 ml of distilled water and centrifuged for 5 min at 3300 rcf. ~70 ml of viral supernatant was added to the upper chamber of each filter cartridge and centrifuged for 30 min at 3300 rcf/4 °C. Flow-through was discarded. For volumes greater than 70 ml, an additional spin was performed. Concentrated viral supernatant was recovered by removing the upper filter cartridge, inverting it, and placing on top of the small collection cup. It was then centrifuged for 2 min at 1000 rcf/4 °C.

Ultracentrifugation was then used to pellet the lentiviral particles and resuspend them in a small volume (typically 60–70 μl). Beckman Ultra-Clear 13 × 51 mm ultracentrifuge tubes were sanitized (Beckman; 344057) by filling with 70% ethanol. After ~15 min, they were rinsed several times with sterile PBS and left to dry under the hood. Concentrated viral supernatant was then transferred to ultracentrifuge tubes. The collection reservoirs were rinsed with viral resuspension buffer (VRB—20 mM Tris pH 8.0, 250 mM NaCl, 10 mM MgCl_2_, 5% sorbitol) and added to ultracentrifuge tubes. The total volume was brought to ~4 ml with VRB. The contents were mixed before adding a sucrose cushion.

A sterile 20% sucrose cushion was added to the bottom of the ultracentrifuge tubes by pipetting 500 μl directly to the bottom of the tube. Ultracentrifuge tubes were then transferred into the rotor buckets and sealed. Each bucket was weighed on a balance. Weights were then equalized with VRB. Buckets were then placed in an MLS 50 rotor of Beckman Optima UltraCentrifuge and ultracentrifuged for 1 h 30 min at 162502 rcf (45000 rpm) at 4 °C. Ultracentrifuge tubes were removed under the hood and the supernatant decanted. The tubes were left inverted for 2 min to remove excess liquid. Sixty microliters of VRB was then added, with the tubes covered with parafilm to minimize evaporation, and left to rest on ice for up to 1 h to ensure the entire pellet is resuspended. The mix was then transferred to 1.5 ml tubes and centrifuged for 5 min at 2000 × g 4 °C to pellet insoluble debris. It was then aliquoted into cryovials as desired, snap frozen in liquid nitrogen and stored at − 80 °C.

### FACS analysis and titre calculation

Analysis of transduced cells was performed using an LSRFortessa X-50 (BD) at the FHCC Flow Cytometry Core Facility. 150,000 lenti-X 293T cells were infected with serially diluted concentrated lentivirus; one well per plate had its cells counted to calculate an accurate initial cell number. To infect cells, 500 μl diluted viral supernatant was used per well. Cells were left for 48 h before FACS analysis. The cells were then washed gently with PBS and trypsinized with TrypLE into single cells. The cells were then collected and filtered through 35 μm FACS tube filters (Corning; 352235). 1.0 μg/ml DAPI was added to stain dead cells before proceeding to FACS analysis. Duplicate samples were performed per virus dilution. Titre, calculated as transducing units per ml (TU/ml), was calculated using samples whose florescence level in living single cells was above 1% and below 20%, in order to generate a titre from within the linear and detectable range.

The concentrated virus used for microinjections was found to be within the same order of magnitude of concentration: pSGEP-luci = 9.2 × 10^7^ TU/ml; pSGEP-p53_843 = 1.3 × 10^8^ TU/ml; pSGEP-p53_1558 = 6.5 × 10^7^ TU/ml; and H2B-RFP = 7.1 × 10^7^ TU/ml. This finding is in line with the fact they were produced at the same time using an identical protocol (see above).

### shRNA design and shRNA vector

The design of the miRE shRNA hairpins was performed using the SPLASH algorithm (http://splashrna.mskcc.org/) [27]. The vector used to clone shRNA hairpins into was the pSGEP vector donated by the Zuber lab [28]. This vector uses the spleen focus-forming virus (SFFV) promoter to drive transcription of the inserted vector. 22-mer guides: Trp53_842 TTACACATGTACTTGTAGTGGA, Trp53_1558 TGAGATTTCATTGTAGGTGCCA, and Renilla luciferase TAGATAAGCATTATAATTCCTA.

### Tissue processing and sectioning

For cryosectioning, the intestine was placed inside moulds with Neg50 embedding medium gently overlaid. The mould was then frozen solid atop a − 80 °C metal freezing block. Frozen blocks were sectioned using a cryostat (Leica, CM1950) at 25 μm thickness, with thin sliced sections collected on SuperFrost plus slides for later staining or imaging. Blocks and slides were stored at − 80 °C.

### Fluorescent imaging

#### Stereoscopic imaging of native intestine

Intestinal tissue was excised and washed in 30 ml cold PBS by shaking 5–10 times. Small intestine tissue was folded into 3 equal parts and cut, creating the duodenum (1st third), jejunum (2nd third) and ileum (3rd third) used for subsequent analysis. These tissue segments were kept in cold PBS to prevent degradation, with only one mouse processed at a time for the same reason.

All stereoscopic imaging was performed using a Zeiss Axio Zoom.V16 microscope. Each tissue segment had its reverse side fully imaged in individual images to visualize fluorescent crypts. These were then later stitched together to provide an overview of regional transduction (see ImageJ analysis below).

#### Confocal fluorescent imaging

All confocal imaging was performed using a Zeiss LSM700 laser scanning microscope. DAPI at 1 μg/ml was used as a DNA staining agent for contrast.

### ImageJ analysis

Analysis was performed blind using Fiji (ImageJ version 1.52n). Original Zeiss CZI format images were converted into TIFF format images for Fiji analysis. In order to perform analysis on each intestinal region a large composite image was created. This composite image consists of smaller individual images that possessed small overlaps, which could then be overlayed and joined with the consecutive image.

Generation of composite images:

1. Canvas size function. Canvas size was readjusted to fit all the images of a tissue.
2. Images were added individually as overlays. The images were added with opacity set at 40% and then added to the ROI manager.
3. The image was then aligned with previous image. Once fully aligned, the opacity was reverted to 100% before the composite image was flattened.
4. A new image overlay is then added to the flattened image and the process repeated.

To assess the number and area of transduced crypt fields directly, regions of interest (ROIs) were manually created around transduced crypt fields on composite images (described above) and analysed by Fiji’s ‘measure’ function in the ROI manager.

ROI analysis:

1. Fields of transduced crypts were selected using polygon selection tool and added to the ROI manager. Only fields on > 10 crypts were selected as defined targeting events; neighbouring fields within ~1–5 crypt width distances were also selected as broken-off crypts that possibly have migrated away from the initial field (splintering). However, these splintered crypts could have resulted from an extra targeting event, which cannot be excluded (see above). Interjacent, untransduced crypts were not included in the calculation of transduced crypt fields.
2. Set scale function. Zeiss images were used as reference to set the scale of TIFF images in Fiji.
3. The measurement function was used to record the ROI data of area size of individual fields.
4. Adjoining fields within 1-5 crypt width distances were regarded as one coherent crypt field. The number of transduced crypt fields and the area of these fields were calculated according to this definition (not including interjacent, untransduced crypts).
5. Only intestinal regions with a minimum number of crypt fields (> 10 crypts fields for duodenum and jejunum, > 5 for ileum) were analysed for physical parameters. This was done to provide statistical significance and reproducibility.

To calculate the total area of transduction for each intestinal region, each respective region had its total area outlined as a ROI, the sum of the transduced crypt field ROIs described above was then divided by this total area and expressed as a percentage.

Supplementary figure 2 shows the most transduced mouse. The transduction area measurements for supplementary figure 2 were calculated slightly differently as the transduced fields could not be accurately outlined.

Analysis of high transduction tissue:

1. Split channel function. Discard Green and Blue Channels.
2. Threshold function. Manually adjust the threshold to best represent the transduced areas.
3. Manual demarcation of whole tissue using polygon selection tool and ROI manager.
4. Measurement function. Record results of threshold area.

Crypt number analysis:

To assess the number of individual crypts within each transduced crypt field (see above) the crypts were manually counted in ImageJ. The total number of crypts was divided by the number of fields to find the average number of crypts per crypt field.

Crypt area analysis:

To find the average area of individual transduced crypts, 15 individual crypts from 3 different fields per intestine region had their area manually demarcated in ImageJ.

### Statistics

All data was analysed in GraphPad Prism (Version 9.0.1). All data passed D’Agostino & Pearson normality test. *p* < 0.05 was considered to indicate statistical significance. Dot plots show means plus-minus one standard deviation. Raw data is available from GBG upon request.

## Supplementary Information

**Additional file 1.**

**Additional file 2: **Supplementary figure 1. Number of transduced crypt fields in different small intestine regions. The number of transduced fields were combined for sh-p53 and sh-luci viruses for wildtype (WT) and *Apc*^Min/+^ (APCmin) cohorts. Kruskal-Wallis analysis was performed with Dunn’s multiple comparisons test, statistical significance is indicated by the *p*-value = 0.0153. Intestinal regions analysed: duodenum WT *n* = 20, duodenum APCmin *n* = 15, jejunum WT *n* = 17, jejunum APCmin *n* = 14, ileum WT *n* = 16, ileum APCmin *n* = 14.

**Additional file 3:.** Supplementary Figure 2. Highest transduced intestinal tract. Composite images of a lentiviral H2B-RFP (red fluorescence) transduction of the small intestine and colon imaged from non-luminal side (reverse) natively using a fluorescent stereoscope. First: composite image of duodenum; second: composite image of jejunum; third: composite image of ileum; fourth: composite image of colon. Scale bars: 5 mm.

**Additional file 4:.** Supplementary Figure 3. Fluorescent stereoscopic images of transduced intestine at embryonic day E17.0-E18.0 and postnatal day (P)14. Control H2B-RFP virus was injected into the yolk sac cavity of mice at E8.0. Left column, RFP viral fluorescence in the intestine was detected at E17.0-E18.0 by examination using a fluorescent stereoscope: top left, image of duodenum; bottom left, image of jejunum. Scale bars: 1 mm. Middle column, RFP viral fluorescence in the intestine was detected at P14 by examination using a fluorescent stereoscope: top middle, fields of RFP-positive crypts imaged from the underside of the small intestine; bottom middle, fields of RFP-positive colon crypts imaged from the luminal side. Scale bars: 1 mm. Right column, RFP viral fluorescence in the intestine was detected at P14 by examination using a confocal microscope: top right, native cryosection of small intestine; bottom right, native cryosection of colon. Scale bars: 0.2 mm. Colours for confocal images: Blue, DAPI; Green, autofluorescence; Red, H2B-RFP.

**Additional file 5: **Supplementary Figure 4. qRT-PCR of *Trp53* in MEFs after shRNA knockdown. Data are expressed relative to *ACTB* and are given normalised to the *Trp53* levels in non-transduced cells. Fold change was calculated using delta-delta Ct from technical triplicates for replicate cultures of transduced mouse embryonic fibroblasts (MEFs). *n* = 8 repeat cultures of transduced MEFs per group. One outlier was identified in the shRNA_Trp53-842 group (using ROUT method, Q = 1%) and removed from analysis. Data were log-transformed and analysed by one-way ANOVA. Statistical significance is indicated by the *p*-value.

**Additional file 6: **Supplementary Figure 5. Images and quantification of fully and partially transduced polyps in *Apc*^Min/+^ mice. Left: example images of transduced polyps (circled in white dashed line); top, image of a fully transduced polyp; bottom, image of a partially transduced polyp. Scale bars: 1 mm. Right: Quantification of fully and partially transduced polyps in *Apc*^Min/+^ mice, sh-luci (*n* = 2 mice), sh-p53 (*n* = 4 mice; 1 mouse was infected with shRNA_Trp53-1558 and 3 mice infected with shRNA_Trp53-842).

**Additional file 7:.** Supplementary Figure 6. Transduction of endoderm-derived organs from E8.0 endoderm targeting. Example images of H2B-RFP and SFFV-GFP transduced organs imaged natively with a fluorescent stereoscope. Scale bars: 1mm.

**Additional file 8: **Supplementary Table 1. Contingency table of microinjected litters analysed for fluorescence. Column variables: genotype of mice born after microinjection procedure. Row variables: observed fluorescence in the intestine, with at least two regions required to be deemed transduced. Numbers represent the count of mice. Chi-square (and Fisher’s exact) test was applied and a *p*-value >0.9999 was determined.

## Data Availability

The datasets used and/or analysed during the current study are available from the KLR and GBG on reasonable request.
