# Supplementary figures and images for: Lentiviral in situ targeting of stem cells in unperturbed intestinal epithelium
Source: BMC Biol. 2023 Jan 11;21:6. doi: 10.1186/s12915-022-01466-1 (PMC9832770; doi:10.1186/s12915-022-01466-1)

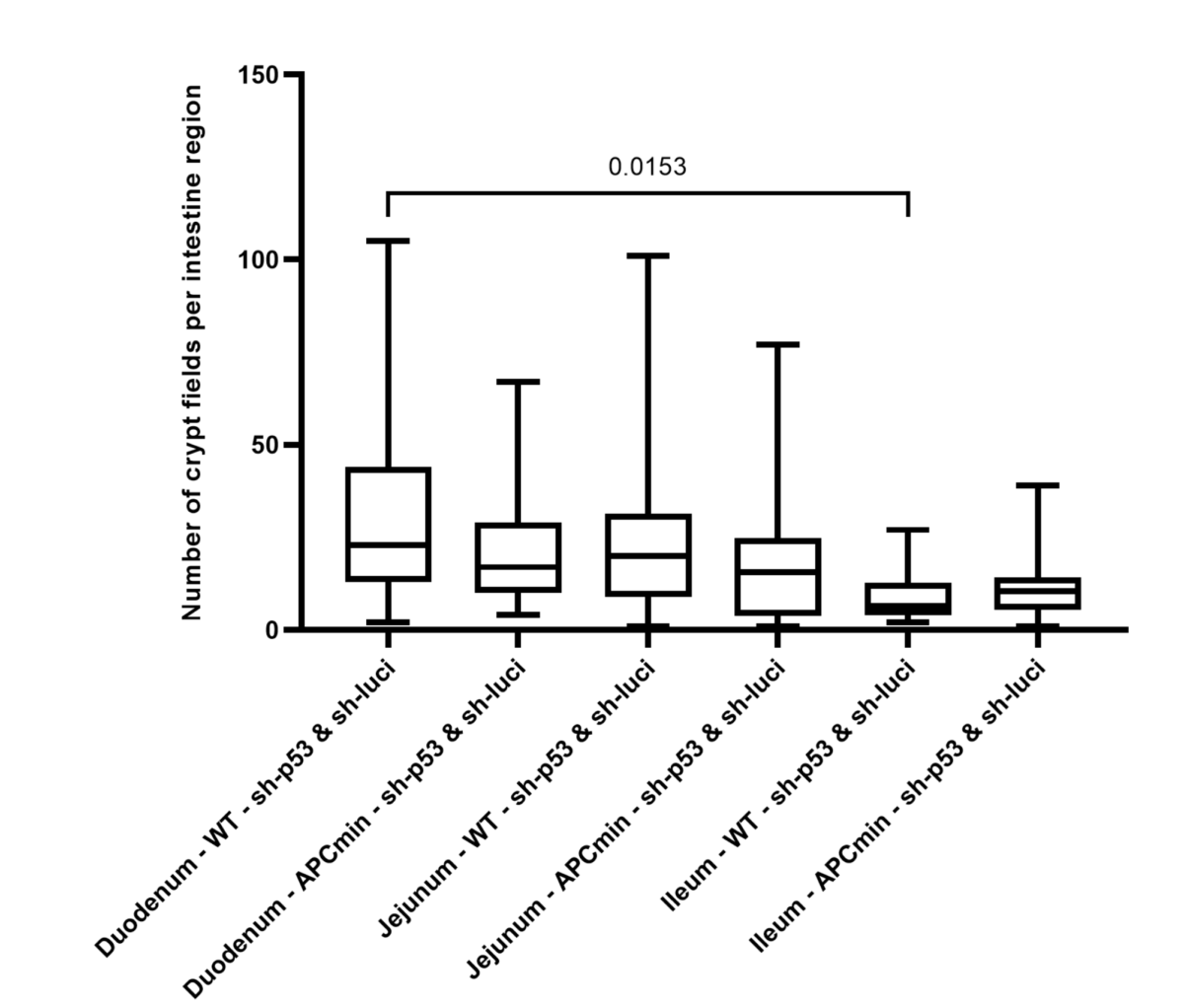

Supplement: Supplementary file 2 — Additional file 2: Supplementary figure 1. Number of transduced crypt fields in different small intestine regions. The number of transduced fields were combined for sh-p53 and sh-luci viruses for wildtype (WT) and ApcMin/+ (APCmin) cohorts. Kruskal-Wallis analysis was performed with Dunn’s multiple comparisons test, statistical significance is indicated by the p-value = 0.0153. Intestinal regions analysed: duodenum WT n = 20, duodenum APCmin n = 15, jejunum WT n = 17, jejunum APCmin n = 14, ileum WT n = 16, ileum APCmin n = 14. [file 12915_2022_1466_MOESM2_ESM.tif]

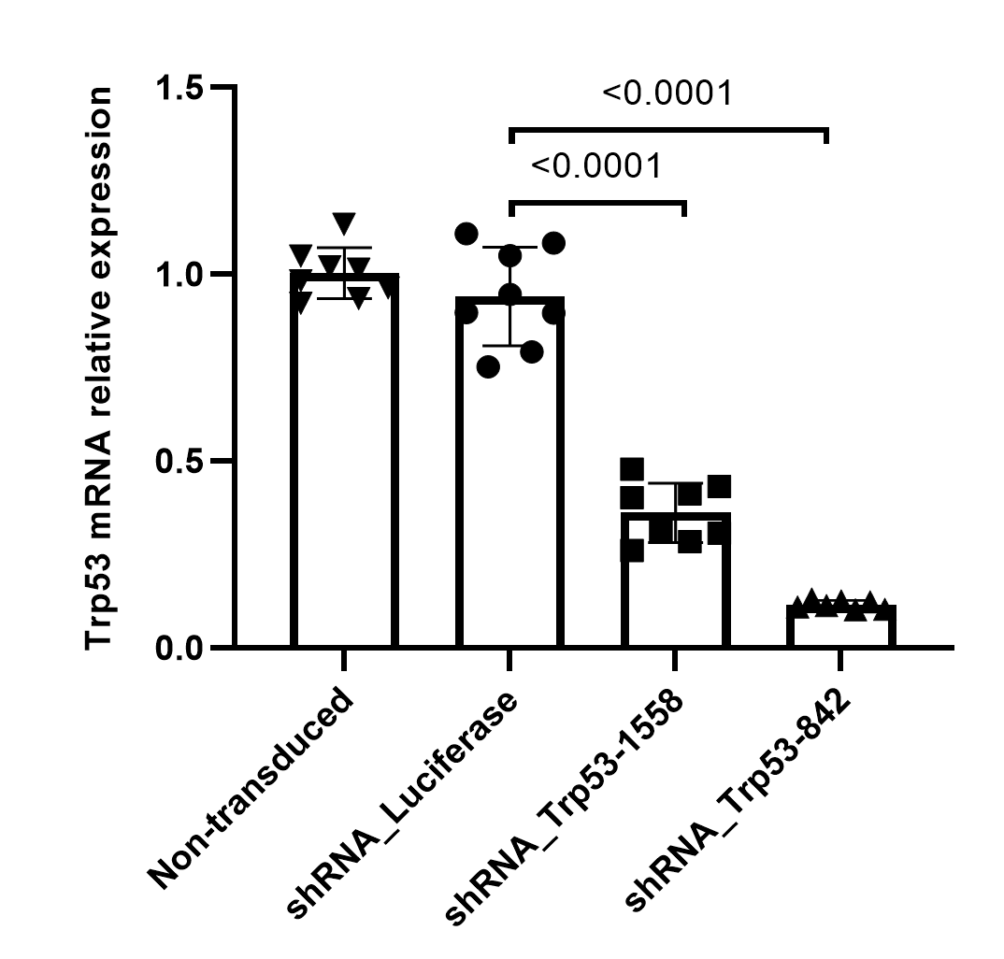

Supplement: Supplementary file 5 — Additional file 5: Supplementary Figure 4. qRT-PCR of Trp53 in MEFs after shRNA knockdown. Data are expressed relative to ACTB and are given normalised to the Trp53 levels in non-transduced cells. Fold change was calculated using delta-delta Ct from technical triplicates for replicate cultures of transduced mouse embryonic fibroblasts (MEFs). n = 8 repeat cultures of transduced MEFs per group. One outlier was identified in the shRNA_Trp53-842 group (using ROUT method, Q = 1%) and removed from analysis. Data were log-transformed and analysed by one-way ANOVA. Statistical significance is indicated by the p-value. [file 12915_2022_1466_MOESM5_ESM.tif]
