# Supplementary material for: Lentiviral in situ targeting of stem cells in unperturbed intestinal epithelium
Source: BMC Biol. 2023 Jan 11;21:6. doi: 10.1186/s12915-022-01466-1 (PMC9832770; doi:10.1186/s12915-022-01466-1)
Supplement: Supplementary file 8 — Additional file 8: Supplementary Table 1. Contingency table of microinjected litters analysed for fluorescence. Column variables: genotype of mice born after microinjection procedure. Row variables: observed fluorescence in the intestine, with at least two regions required to be deemed transduced. Numbers represent the count of mice. Chi-square (and Fisher’s exact) test was applied and a p-value >0.9999 was determined. [file 12915_2022_1466_MOESM8_ESM.docx]

|  | *Apc^M^*^in/+^ | Wildtype | Total |
| --- | --- | --- | --- |
| Transduced | 15 | 21 | 36 |
| Untransduced | 31 | 44 | 75 |
| Total | 46 | 65 | 111 |

**Supplementary table 1**. Contingency table of microinjected litters analysed for fluorescence. Column variables: genotype of mice born after microinjection procedure. Row variables: observed fluorescence in the intestine, with at least two regions required to be deemed transduced. Numbers represent the count of mice. Chi-square (and Fisher’s exact) test was applied and a p-value >0.9999 was determined.
